# Hemorrhagic versus ischemic stroke: Who can best benefit from blended conventional physiotherapy with robotic-assisted gait therapy?

**DOI:** 10.1371/journal.pone.0178636

**Published:** 2017-06-02

**Authors:** Frédéric Dierick, Mélanie Dehas, Jean-Luc Isambert, Soizic Injeyan, Anne-France Bouché, Yannick Bleyenheuft, Sigal Portnoy

**Affiliations:** 1 Forme & Fonctionnement Humain Research Unit, Department of Physical Therapy, Haute Ecole Louvain en Hainaut, Montignies sur Sambre, Belgium; 2 Faculty of Motor Sciences, Université catholique de Louvain, Louvain-la-Neuve, Belgium; 3 « Le Normandy » Rehabilitation Center, Granville, France; 4 Rehabilitation Unit, « Le Richemont », Bioul, Belgium; 5 Institute of Neuroscience, Université catholique de Louvain, Brussels, Belgium; 6 Department of Occupational Therapy, Sackler School of Medicine, Tel Aviv University, Tel Aviv, Israel; Northwestern University, UNITED STATES

## Abstract

**Background:**

Contrary to common belief of clinicians that hemorrhagic stroke survivors have better functional prognoses than ischemic, recent studies show that ischemic survivors could experience similar or even better functional improvements. However, the influence of stroke subtype on gait and posture outcomes following an intervention blending conventional physiotherapy with robotic-assisted gait therapy is missing.

**Objective:**

This study compared gait and posture outcome measures between ambulatory hemorrhagic patients and ischemic patients, who received a similar 4 weeks’ intervention blending a conventional bottom-up physiotherapy approach and an exoskeleton top-down robotic-assisted gait training (RAGT) approach with Lokomat.

**Methods:**

Forty adult hemiparetic stroke inpatient subjects were recruited: 20 hemorrhagic and 20 ischemic, matched by age, gender, side of hemisphere lesion, stroke severity, and locomotor impairments. Functional Ambulation Category, Postural Assessment Scale for Stroke, Tinetti Performance Oriented Mobility Assessment, 6 Minutes Walk Test, Timed Up and Go and 10-Meter Walk Test were performed before and after a 4-week long intervention. Functional gains were calculated for all tests.

**Results:**

Hemorrhagic and ischemic subjects showed significant improvements in Functional Ambulation Category (*P*<0.001 and *P* = 0.008, respectively), Postural Assessment Scale for Stroke (*P*<0.001 and *P* = 0.003), 6 Minutes Walk Test (*P* = 0.003 and *P* = 0.015) and 10-Meter Walk Test (*P* = 0.001 and *P* = 0.024). Ischemic patients also showed significant improvements in Timed Up and Go. Significantly greater mean Functional Ambulation Category and Tinetti Performance Oriented Mobility Assessment gains were observed for hemorrhagic compared to ischemic, with large (*dz* = 0.81) and medium (*dz* = 0.66) effect sizes, respectively.

**Conclusion:**

Overall, both groups exhibited quasi similar functional improvements and benefits from the same type, length and frequency of blended conventional physiotherapy and RAGT protocol. The use of intensive treatment plans blending top-down physiotherapy and bottom-up robotic approaches is promising for post-stroke rehabilitation.

## Introduction

In 2013, the worldwide prevalence of stroke was 25.7 million, with 10.3 million individuals having a first stroke, and about 2 of every 3 first strokes were of ischemic nature [1]. Stroke is a common and disabling worldwide health-care problem. By 2030, there are estimated to be almost 70 million stroke survivors [2].

Though neurorehabilitation is a key part of patient care [3], there remains a lack of evidence indicating which rehabilitation strategies are most beneficial in promoting functional independence in post-stroke patients [4], especially through improvement of standing posture and locomotion.

In recent years, the efficiency of diverse task-oriented training techniques for stroke patients has been demonstrated in several meta-analyses, e.g. body weight-supported treadmill training (BWSTT) [5], circuit class training [6], augmented exercise therapy [7], and automated locomotion therapy [8]. In the latter case, the automation of lower limb movements during locomotion is ensured by electromechanical/ robotic devices, that were developed to help the physiotherapists by increasing the safety, intensity and standardization of non-robotic BWSTT, generate complex multisensory stimulation, provide extensive extrinsic biofeedback to the patient, and reduce working costs [9,10].

The vast majority of randomized controlled trials with small samples (n ≤ 40) of subacute to chronic hemiparetic stroke patients, comparing one of the two widespread robotic-assisted gait therapy (RAGT) systems, namely the Lokomat (Hocoma, Volketswil, Switzerland) or the Gait Trainer (Reha-Stim, Berlin, Germany), with BWSTT or conventional physiotherapy exercises or even a combination of the two approaches, has shown potential of RAGT to facilitate greater functional improvements. Balance [11,12], gait speed [11,12,13], walking ability or endurance [12,13,14], and mobility disability [14] were improved after only 12 to 20 sessions. However, two randomized controlled trials with larger samples (n = 48 and n = 72) raised doubts about the effectiveness of RAGT (12 to 24 sessions) compared to conventional gait training based on BWSTT without exercises in 48 chronic (defined as > 6 months post stroke) ambulatory patients [15] or to BWSTT with exercises and overground walking in 72 subacute (defined as < 6 months post stroke) patients with moderate to severe gait impairments [16].

To date, a usage of RAGT in rehabilitation centers is limited due to: (1) the need for trained personnel, (2) the scheduling availability of the system, (3) the high cost of the technology, and (4) the skepticism of some members of rehabilitation teams [17] that is probably based on lack of clear guidelines about RAGT protocols tailored on patients’ characteristics and history, and motor capacities [18,19]. We believe that the first two limitations can easily be resolved in rehabilitation centers, implementing some organizational adjustments and that the high cost is irrelevant, at least in industrial countries. Indeed, the added cost of delivering robot therapy alongside usual care is lower compared to an intensive therapy alongside usual care [20], and therefore the real financial problem is related to the intensity of the rehabilitation program and not the choice of the rehabilitation strategy.

At the light of all above studies and limiting factors for the development of RAGT, it seems that efficiency of RAGT would be mainly related to a correct identification of the target population [19], in other words: *“is RAGT more suitable for a specific patient group over another*?*”* [15]. Morone et al. [21,22] even suggested to consider an alternate scientific question: *“who may benefit from RAGT*?*”*; this important, unanswered clinical question was the rational for this study. The last updated Cochrane review conducted by Mehrholz et al. [8] on the use of *“electromechanical-assisted training for walking after stroke”*, helped to legitimize this question since it found evidence that RAGT combined with physiotherapy may improve recovery of independent walking in people in the first 3 months after stroke (defined as subacute) but not in people after 3 months (defined as chronic). Moreover, in a recent randomized controlled trial [23], RAGT using Lokomat was more effective than treadmill gait training in improving walking ability, balance and balance confidence in chronic patients (defined as > 6 months).

It is well known that one major source of bias of randomized controlled studies aiming to compare different rehabilitation strategies comes from the natural recovery of stroke, heterogeneous in its nature [3], that might be a confounder for the interpretation of functional post-stroke improvements. Other factors that may influence interpretation are severity of paralysis [24], level of activities of daily living (ADL) [25,26], anatomic localization of the lesion [27], affected cerebral hemisphere [28,29], extent of subsequent recovery [30,31], age [32,33], gender [34], rehabilitation treatment plan [34–36], as well as lesion etiology [30,31,37].

The rationale for studying the influence of lesion etiology, namely hemorrhagic or ischemic, on rehabilitation outcomes is based on the different molecular pathophysiologic cascades [38,39] underlying brain injury and possibly different cerebral and functional implications [40]. Although it is generally believed that hemorrhagic stroke survivors have better neurological and functional prognoses than ischemic stroke survivors, a recent study highlighted that data are mixed [40], with studies indicating better results in hemorrhagic [30,37,41,42], in ischemic [43] or even no differences between the two subtypes [44]. Finally, some studies showed that specific gait characteristics were associated to lesion etiology [45,46], strongly suggesting that subtype of stroke should be considered as a factor in gait assessment and rehabilitation protocols and therefore in establishing clinical studies focusing on RAGT.

To the best of our knowledge, no study, with a balanced number of stroke subtypes, explored the influence of lesion etiology on an extensive therapy plan blending conventional physiotherapy and RAGT. The objective of this study was therefore to compare gait and posture outcome measures between ambulatory hemorrhagic and ischemic patients who received a similar well-standardized 4 weeks’ intervention blending conventional physiotherapy and RAGT. The devices which are currently used for RAGT can be differentiated into end-effector devices, which move the feet of the subject, and exoskeleton devices, which move the hips and knees. The RAGT system used in our study is an exoskeleton device: the Lokomat. Since studies that tested the effect of end-effector devices usually include subjects with close to independent walking [47], we will only compare the effectiveness of our treatment with clinical studies realized with exoskeleton devices, and more specifically with the Lokomat.

We hypothesized that an extensive treatment program will greatly improve functional outcomes of hemorrhagic patients compared to ischemic patients since: (1) most previous studies conducted on post-stroke patients showed that those of hemorrhagic nature exhibited greater functional improvements; and (2) the neurological deficit caused by mechanical compression of the brain tissue improves as the hematoma resolves as well as ischemia in the penumbra area that surrounds it [42]. To reduce potential bias, the groups of stroke patients were matched by age, gender, side of hemisphere lesion, stroke severity based on dependence in ADL, and locomotor impairments based on self-selected gait speed at baseline.

## Materials and methods

### Participants

This retrospective study included subjects presenting hemiparesis from a first supratentorial space-occupying stroke of either ischemic or hemorrhagic origin. Both the assessment method and training program are current procedure of the rehabilitation center. This study was approved by a local ethics committee (Centre Hospitalier Avranches, Granville). Due to retrospective nature and the lack of subject interaction, this study did not require informed consent.

From a prospectively-maintained medical report database, we identified 40 subjects who were selected over a 3-years period. Inclusion criteria were as follows: adult patients (at least 18 years old) following a unilateral stroke occurring in the past year at most (time since stroke < 52 weeks), admitted for inpatient rehabilitation, enrolled for the first time in an intervention based on RAGT, able to understand and follow verbal instructions, ambulatory: having a Functional Ambulation Category (FAC) ≥ 1, and a gait speed slower than 0.8 m s^-1^, i.e.10-Meter Walk Test (10MWT) higher than 12.5 s, limiting the study group to household or limited community walkers [48].

The subjects were divided into two groups, according to their stroke subtype: a hemorrhagic group (HG, n = 20) and an ischemic group (IG, n = 20), as classified using CT or MRI. Depending on the Trial of Org 10172 in Acute Stroke Treatment. (TOAST) classification [49], the IG was composed of 10 subjects with large-artery atherosclerosis, 5 with small-artery occlusion, and 5 with cardioembolism.

The groups were matched by age (± 3 years), sex, side of hemisphere lesion, and stroke severity using the 10-item Barthel Index scale (BI) [50], scoring 0 to 100 with 5-point increments [51]. Subjects were included only with a BI score ≤ 60 [52], indicating a severe dependence in ADL. Subjects were further matched based on their self-selected gait speed at baseline; those who walked at a speed ≤0.5 m s^-1^ (10MWT ≥20 s) were classified with severe locomotor impairments and those who walked at a speed >0.5 m s^-1^ (10MWT <20 s) with moderate locomotor impairments [15]. Demographic and clinical characteristics of subjects at baseline are reported in Table 1. Subjects without brain lesion on CT scans or MRI were excluded to avoid enrolling transient ischemic attack patients. The matching was confirmed statistically so that there were no significant differences between HG and IG subjects (Table 1). Age differences were explored using a paired t-test, proportion of stroke conditions and proportion of patients with previous Achilles tenotomies or *triceps surae* botulinum toxin injections using χ^2^ tests.

**Table 1:** Demographic and clinical characteristics of patients at baseline in the hemorrhagic and ischemic groups.

	Hemorrhagic (HG)	Ischemic (IG)		*P*
Patients, n	20	20		
Age, years	55.9 ± 12.3	56.3 ± 11.2		0.926†
Gender, n (male/female)	9/11	9/11		
Side of hemisphere lesion, n (left/right)	12/8	12/8		
Barthel Index (BI)	47.50 (37.5–55.0)	47.50 (37.5–55.0)		
Functional Ambulation Category (FAC)	3.5 (1–4)	2.5 (1–4)		0.782#
Postural Assessment Scale for Stroke (PASS)	30.5 (29–33)	30.5 (26–31)		0.312#
Tinetti POMA (TT)	16.5 (11–18)	17.5 (15–18)		0.130#
6 Minutes Walking Test (6MWT) (m)	110.80 ± 64.2	123.70 ± 90.2		0.938†
Time Up and Go (TUG) Test (s)	39.95 ± 19.6	48.95 ± 38.2		0.675†
10 Meters Walking Test (10MWT) (s)	32.25 ± 16.4	44.85 ± 34.2		0.134†
Locomotor impairments, n (moderate/severe)	7/13	7/13		
Stroke condition, n (subacute/chronic)*	4/16	5/15		0.705‡
Time since stroke (weeks)	28.7 ± 13	29.4 ± 12		0.853†
Achilles tenotomy, n	8	3		0.077‡
Triceps surae botulinum toxin injection, n	5	5		

Tinetti POMA: Tinetti Performance Oriented Mobility Assessment.

Data are presented as mean ± SD for age, 6 MWT, TUGT and 10MWT.

Data are presented as median (q1-q3) for BI, FAC, PASS, and Tinetti POMA.

* Subacute defined ≤ 3 months after stroke and chronic defined > 3 months after stroke.

† *P* value derived from paired t test.

# *P* value derived from Wilcoxon signed rank test.

‡ *P* value derived from χ^2^ test.

### Intervention

All subjects participated in a 4-week standardized neurorehabilitation intervention blending RAGT (Lokomat, Hocoma, Volketswil, Switzerland) and conventional physiotherapy.

Each subject received a 60 minutes RAGT session and additional 45 minutes’ physiotherapy, 5 days a week for 4 weeks. Each RAGT session comprised of a maximum of 30 minutes of effective RAGT and the other 30 minutes were spent on mounting, dismounting, and adjustment of the system. Initial walking speeds were 0.28 m s^-1^ for participants with severe locomotor impairments and 0.44 m s^-1^ for participants with moderate locomotor impairments. These speeds were not increased during the first session to allow the participants to get used to the Lokomat. During each session, the speed of the treadmill was set to the maximum speed tolerated by the subjects and which did not make them uncomfortable, up to a maximum of 0.83 m s^-1^. Elastic straps were used to assist toe clearance. The guidance force (GF) of the hip and knee motor drives of the hemiparetic lower limb was provided during both stance and swing phases and was gradually reduced, even on the first session, depending on the subject’s needs, from 100% to 0%. Subjects were given real-time visual feedback of hip and knee torques during the training session and the physiotherapists verbally motivated the subjects to actively move their lower limbs. Body weight support (BWS) was set at 40% during the first session and not decreased during this first session. During each session, the level of BWS was gradually reduced to 0%, as tolerated by the subjects, in increments of 5 to 10% per session. In addition, physiotherapists strongly discouraged the subjects to use the handrails. The goal for each participant was to walk for a total of 30 minutes in the Lokomat under no BWS, at 0.83 m s^-1^ and a GF of 0%. The sequence of variable progression was: (1) treadmill speed; (2) time duration; (3) GF; and (4) BWS [53]. The Lokomat training parameters were collected at first and last session to study the progression of the participants.

The conventional physiotherapy was based on neurophysiological concepts such as the Bobath approach [54]. Strategies utilized in these sessions emphasized general bilateral and tri-dimensional movements required for turning, rolling, kneeling, sitting, standing, and so on, as well as integration of the selective movement in functional activity and exercise for improving balance. The key aspects of clinical practice of the Bobath approach are related to the identification of movement deficits, the analysis of movement and its quality, the use of afferent sensory information from multiple sources and facilitation, and minimizing motor solutions that incorporate motor compensations [55].

### Gait and posture outcome measures

Measurement of gait and posture outcomes were performed before and after the intervention by means of six standardized tests: Functional Ambulation Category (FAC) [56], Postural Assessment Scale for Stroke (PASS) [57], Tinetti Performance Oriented Mobility Assessment (TT) [58], 6 Minutes Walk Test (6MWT) [59], Timed Up and Go (TUG) [60], and 10-Meter Walk Test (10MWT) [61].

FAC assesses functional ambulation and is rated on a ‘0’ (nonfunctional) to ‘5’ (independent) scale. PASS is scored between ‘0’ (poor balance) and ‘36’ (good balance); it assesses ability to maintain stable postures as well as balance during changes of position. TT can reach a score of 28 points which will represent an independent patient; it comprises of a gait component (12 points), balance component (16 points), and specifies that a score below 19 indicates risk of fall. 6MWT was measured at the maximal possible walking speed; it assesses the distance in meters walked over 6 minutes as a sub-maximal test of aerobic capacity or endurance. TUG was measured in seconds; it assesses mobility, balance, and walking ability. 10MWT was performed at the preferred walking speed and calculated as the average of the three trials; it assesses walking speed in meters per second over a short duration.

During all the tests, the subjects could use walking aids, e.g. a dynamic orthosis or a tripod cane. Where a dynamic orthosis was used before the neurorehabilitation intervention, the subject was requested to wear it during the post-intervention evaluation. Both assessments were systematically carried out by the same examiner (S.I.).

For the 6MWT, TUG, and 10MWT, the absolute functional gains were calculated as the difference between “after treatment score” and “baseline score”. Relative functional gain percentages were calculated for FAC, PASS, and TT tests as [(“after treatment score”–“baseline score”) / (“maximum score”–“baseline score”)] x 100 [62].

### Statistical analyses

All statistical analyses were performed using SigmaPlot software (v. 11.0, Systat software, San Jose, CA). All data were tested for normal distribution using the Shapiro-Wilk test. Since FAC, PASS, and TT are ordinal data, results of the tests are presented as medians (q1–q3). The Wilcoxon signed rank test was used in these cases to test for significant difference between the baseline (IN) and after intervention (OUT) scores. The 6MWT, TUG, and 10MWT were normally-distributed so the results are presented as means (± SD). To ascertain similar conditions at IN between the two groups, the results of the gait and posture baseline tests were compared between HG and IG groups. There were no significant differences (Table 1) in FAC, PASS and TT scores (Wilcoxon signed rank test) or 6MWT, TUG, and 10MWT scores (paired t-test). The paired t-test was used in these cases to test for significant difference between the IN and OUT scores. Statistical differences between the Lokomat training parameters (speed, BWS, and GF) in HG and IG at IN and OUT were computed using t-tests. Absolute and relative functional gains were normally-distributed and the unpaired t-test was used to compare between HG and IG. Correlations between the time since stroke and the difference between IN and OUT results were calculated for all the clinical tests, to study the potential relationship of this variable on the results. Effect size *dz* (Cohen’s d) was computed for paired and unpaired t-tests using G*Power software (v. 3.1). For all tests, statistical significance was set at *P* < 0.05.

## Results

### Lokomat training parameters

**Fig 1:**
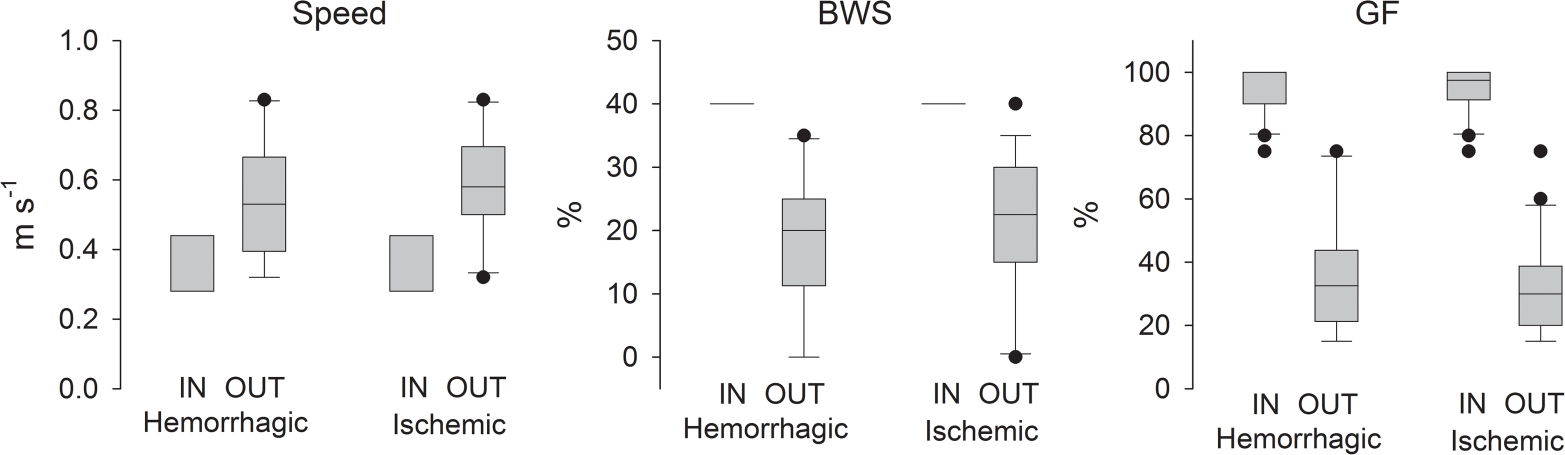
Results for Lokomat training parameters for hemorrhagic and ischemic groups at baseline (IN) and after the intervention (OUT). BWS: body weight-support, GF: guidance force.

The results of the treadmill speed, BWS, and GF parameters, used for the Lokomat training obtained for both HG and IG at baseline (IN) and after the intervention (OUT), are presented in Fig 1. At the end of the intervention, all participants reached the maximum walking time duration of 30 minutes No significant differences of training parameters were observed between HG and IG at IN and OUT.

### Gait and posture results

**Fig 2:**
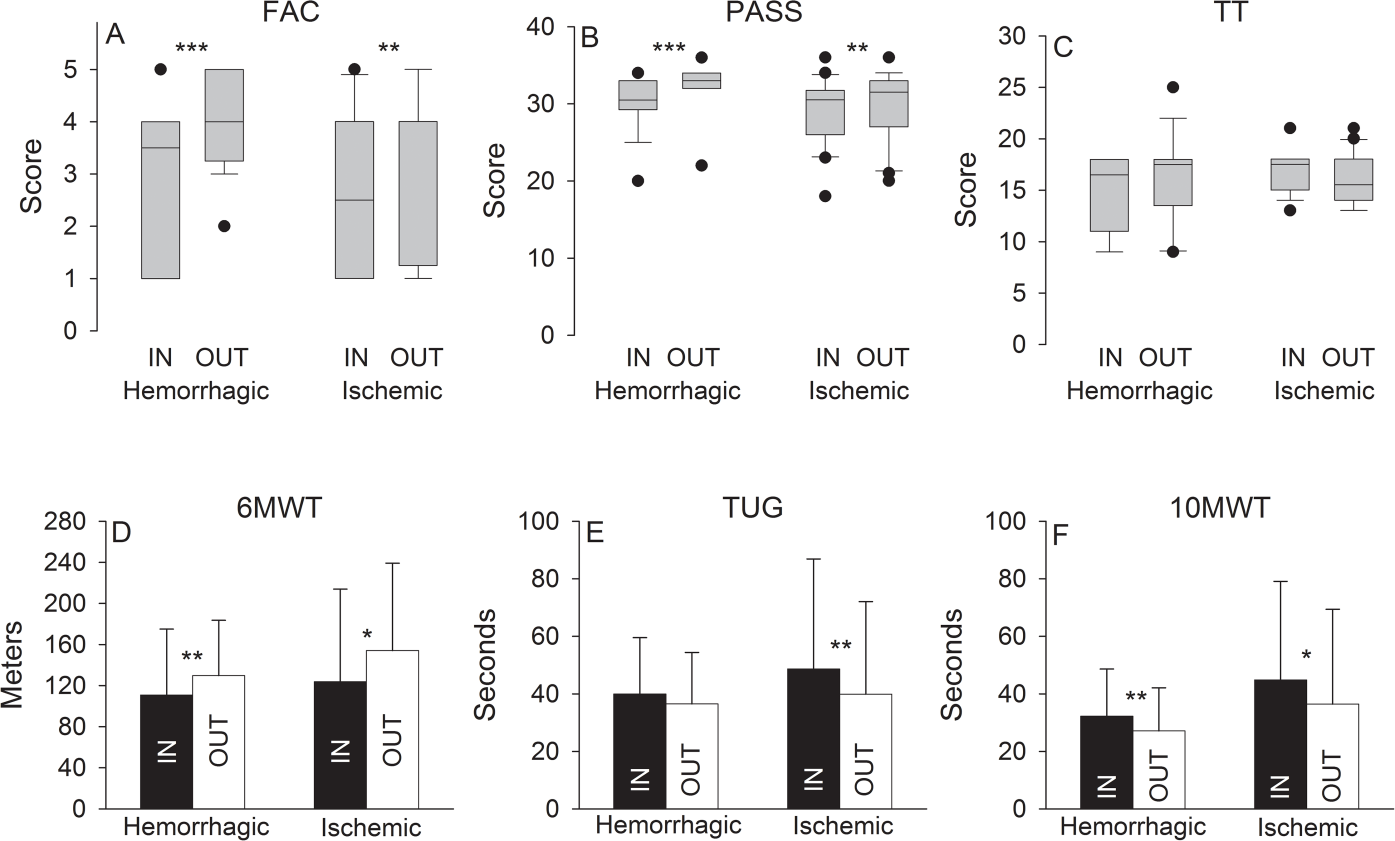
Results for gait and posture for hemorrhagic and ischemic groups at baseline (IN) and after the intervention (OUT). (A) FAC: Functional Ambulation Category, (B) PASS: Postural Assessment Scale for Stroke, (C) TT: Tinetti Performance Oriented Mobility Assessment, (D) 6MWT: 6 Minutes Walk Test, (E) TUG: Timed Up and Go, (F) 10MWT: 10-Meter Walk Test.

The gait and posture results obtained for both HG and IG at baseline (IN) and after the intervention (OUT), are presented in Table 2 and Fig 2. After four weeks, the HG showed significant improvements in all but the TUG and the TT and the IG showed significant improvements in all but the TT (Table 2 and Fig 2). Specifically, the percentage of subjects who had a FAC level ≥4 increased from 50% at IN to 75% at OUT in the HG and from 45% to 55% in the IG. Forty % of HG subjects had a FAC increase of more than 1 point versus only 15% of IG subjects. The percentage of subjects who had a PASS score above 30 increased from 50% to 95% at OUT in the HG and was constant at 55% for IG. TT score increased in 30%, was unchanged in 55%, and was reduced in 15% of the HG at OUT, while in the IG the TT score increased in 20%, was unchanged in 50% and reduced in 30%. Overall, the median TT score in IG was reduced in 2 points at OUT. All HG subjects had a TT score <19 at IN versus 80% at OUT; 15% had a score between 19 and 24, and the last 5% a score ≥25, while 95% of subjects had a TT score <19 at IN versus 85% at OUT; and the last 15% had a score between 19 and 24. Heighty-five % of HG subjects had an unchanged or increased score and 80% of IG subjects had an unchanged or decreased score. In the 6MWT, the HG increased its mean distance from 111 m to 130 and from 0.31 m s^-1^ to 0.37 in the 10MWT, while the IG increased its mean distance from 124 m to 154 in the 6MWT and from 0.22 m s^-1^ to 0.27 in the 10MWT. Finally, TUG was significantly reduced in 9.05 s in the IG at OUT with 70% of subjects showing gains greater than 3.16 s. On the contrary, a non-significant 3.45 s difference exist for HG with 55% of subjects showing gains greater than 3.16 s.

**Table 2:** Comparison of posture and gait results at baseline and after intervention and the effectiveness or functional gains.

	Hemorrhagic (HG)					Ischemic (IG)				
	Baseline (IN)	After (OUT)	W/ t	*P*	*dz*	Baseline (IN)	After (OUT)	W/ t	*P*	*dz*
FAC	3.5 (1–4)	4 (3.5–5)	120.0	**< 0.001**		2.5 (1–4)	4 (1.5–4)	36.0	**0.008**	
PASS	30.5 (29–33)	33.0 (32–34)	120.0	**< 0.001**		30.5 (26–31)	31.5 (27–33)	89.0	**0.003**	
TT	16.5 (11–18)	17.5 (14–18)	24.0	0.164		17.5 (15–18)	15.5 (14–18)	-29.0	0.160	
6MWT (m)	110.80 ± 64.2	129.80 ± 53.8	-3.35	**0.003**	0.32	123.70 ± 90.2	154.10 ± 85.1	-2.68	**0.015**	0.35
TUG (s)	39.95 ± 19.6	36.50 ± 17.9	1.53	0.143	0.18	48.95 ± 38.2	39.90 ± 32.1	3.22	**0.005**	0.25
10MWT (s)	32.25 ± 16.4	27.10 ± 15.0	3.77	**0.001**	0.33	44.85 ± 34.2	36.40 ± 33.0	2.45	**0.024**	0.25

FAC: Functional Ambulation Category.

PASS: Postural Assessment Scale for Stroke.

TT: Tinetti Performance Oriented Mobility Assessment.

6MWT: 6 Minutes Walking Test.

TUG: Time Up and Go Test.

10MWT: 10 Meters Walking Test.

Significant values are in bold; W value of Wilcoxon signed rank test; t value of paired t-test.

*dz*: effect size.

### Functional gains

The relative and absolute functional gains of both IG and HG in the gait and posture tests are depicted in Table 3. Two significant functional gains differences were observed between HG and IG. First, the mean FAC gain was 30% higher in HG compared to IG (t = 2.46, *P* = 0.019). Second, the mean TT gain was 15% higher in HG compared to IG (t = 2.04, *P* = 0.048).

**Table 3:** Relative and absolute functional gains between hemorrhagic and ischemic.

	Hemorrhagic	Ischemic	t	*P*	*dz*
FAC	55.7 ± 37%	26.1 ± 36%	2.46	**0.019**	0.81
PASS	36.5 ± 30%	21.1 ± 22%	1.82	0.077	0.58
TT	9.1 ± 28%	-6.4 ± 18%	2.04	**0.048**	0.66
6MWT (m)	19.05 ± 25.4 m	30.40 ± 50.8 m	0.80	0.377	0.28
TUG (s)	3.45 ± 10.1 s	9.05 ± 12.6 s	2.41	0.129	0.49
10MWT (s)	5.15 ± 6.1 s	8.45 ± 15.4 s	0.79	0.379	0.28

FAC: Functional Ambulation Category

PASS: Postural Assessment Scale for Stroke

TT: Tinetti Performance Oriented Mobility Assessment

6MWT: 6 Minutes Walking Test

TUG: Time Up and Go Test

10MWT: 10 Meters Walking Test

Significant values are in bold; t value of unpaired t-test

*dz*: effect size

### Correlations between time since stroke onset and results

**Fig 3:**
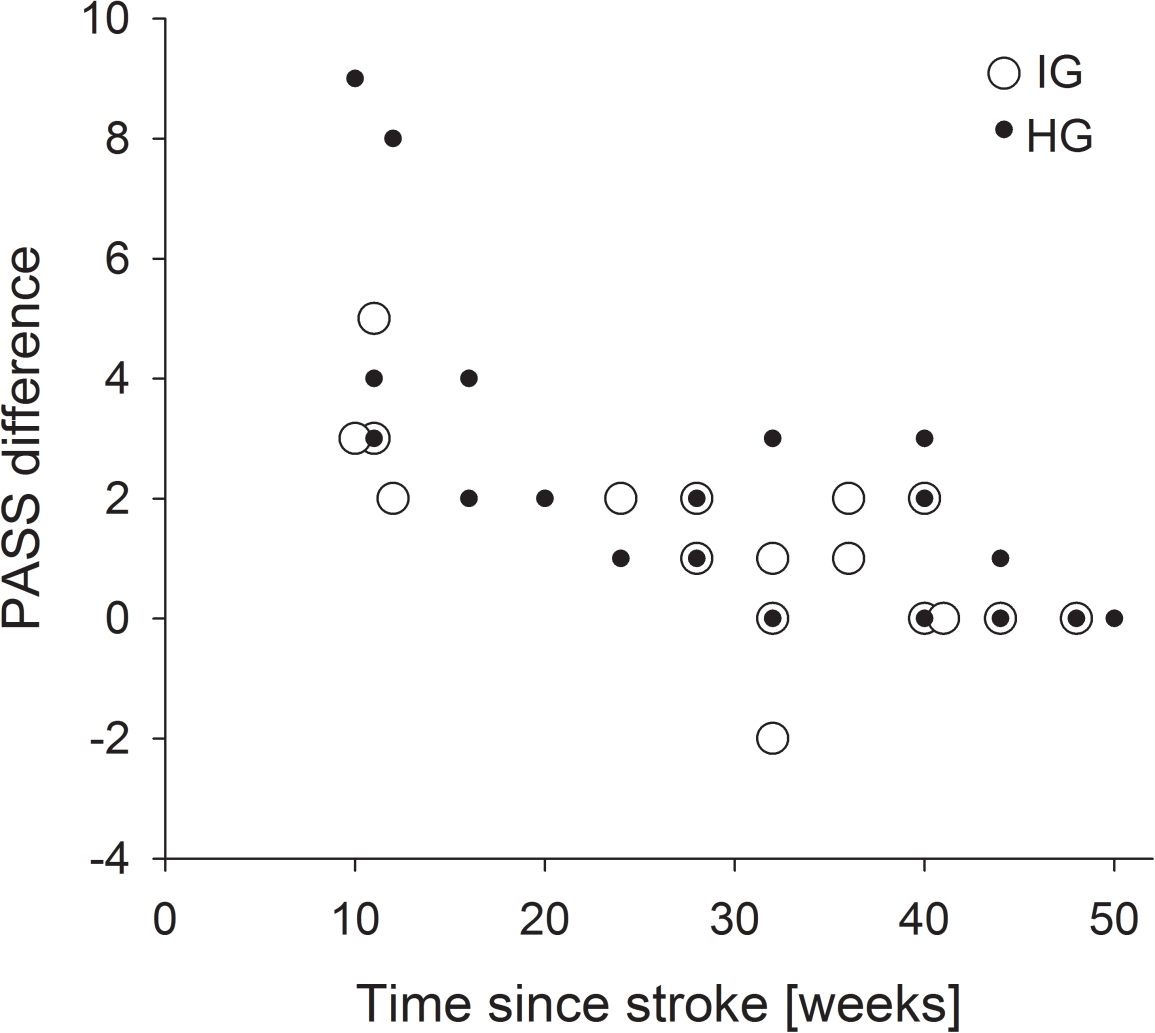
Relationship between time since stroke and PASS difference (OUT score–IN score) for HG (hemorrhagic) and IG (ischemic).

A significant negative correlation between PASS difference and the time since stroke was observed for HG (r = -0.711, *P* < 0.001) and IG (r = -0.710, *P* < 0.001) and is presented in Fig 3. Correlations between FAC, TT, TUG, and 10MWT were not statistically significant.

## Discussion

This is the first study to compare gait and posture outcome measures between ambulatory HG and IG that went through RAGT. Overall, though the pathophysiology of these subtypes of strokes is very different, both groups exhibited quasi similar functional improvements following RAGT. Hemorrhagic stroke initially results from a rupture of cerebral blood vessels and the creation of hematoma associated with a mechanical compression of the brain tissue, while ischemic stroke is the result of development of thrombi and/ or emboli leading to blockages in a cerebral artery resulting in a deficiency of oxygen. Nevertheless, both stroke subtypes could ultimately result in ischemic injuries [40] since blood pressure reduction treatment could results in ischemic insult to perihematomal penumbral lesions surrounding the hemorrhage area [63]. This phenomenon may possibly account for the similarity between groups, demonstrated in our findings. Similar results were previously reported by Perna and Temple [40] who showed that hemorrhagic and ischemic groups of stroke patients, within 6 months post stroke, experienced similar functional levels across all three Mayo Portland Adaptive Inventory-4 domains, however no RAGT intervention was included in their study. Another explanation for the absence of differences between groups could be related to specific combination between the RAGT and conventional physiotherapy protocols. The physiotherapy protocol chosen for this study was based on the Bobath approach that can be classified a technique based on available neurophysiological knowledge [64] and is probably the most widely used approach in Europe. It is also a bottom-up approach that act on the distal physical level (bottom) aiming at influencing the neural system (top) [19]. Opposite to the passive role of the patients implied in neurophysiological techniques, RAGT is a top-down and motor learning approach stressing active patient involvement [19].

Regarding clinical implications, these results show that both groups benefit from the same type, length and frequency of RAGT. We therefore conclude that rehabilitation teams should refrain from indulging the general belief that hemorrhagic stroke survivors could experience better functional improvements than ischemic patients, at least when other main possible confounders are considered.

We were not able to match the groups according to time since stroke. However, no significant differences were found between the mean number of weeks and the proportion of patients in subacute and chronic phases between the groups, with time post stroke of the patients ranging between 10 and 50 weeks in HG and between 10 and 48 in IG. The only variable that significantly correlated with the time since stroke was the PASS score difference, indicating a specific recovery effect of RAGT on balance during the early weeks after stroke. This result is in accordance with Chisari et al. [65] who observed a correlation between the increment of Berg Balance Scale (BBS) score and the elapsed time from the stroke event after RAGT, however the sample used in their study was different, as the time since stroke ranged between 2 and 72 months. To our knowledge, this study is the first to include PASS as an outcome measurement after RAGT with an exoskeleton device. It is of interest in hemiplegic stroke subjects because the monopodal stance is a fundamental stage for the acquisition of independent gait. PASS includes items not assessed by the BBS, such as the ability to roll into a lying position, so it is less likely to have a floor effect [66], and demonstrates better psychometric properties than the BBS [67]. Unfortunately, only the BBS score was formerly assessed, showing both significant [11,16] and non-significant [15] balance improvements following RAGT. In this study, we found significant posture and balance improvements via the PASS scores that increased from a median of 30.5 to 33.0 in HG and from 30.5 to 31.5 in IG (Table 2). Even if statistically significant, this small increase of 1 to 2.5-points on a 36-point scale, must be interpreted cautiously since no minimum detectable change (MDC) or minimal clinically important difference (MCID) values were previously established.

Reduced static and dynamic balance is a common motor impairment after stroke and a major cause of falls and fall-related injury. Interestingly, although there were no intra-group significant differences in TT scores following the intervention, significant inter-group differences were found (Table 2). These inter-group differences are attributed to an average increase in TT scores in the HG, as opposed to an average decrease in TT scores in the IG: 85% of HG subjects had an unchanged or increased score and 80% of IG subjects had an unchanged or decreased score. We do not believe that a 2-points decrease on this 28-point scale points had a real clinical meaning and that the significant difference in TT gains should be considered with caution. However, in the HG, 20% of the subjects increased their TT score ≥19, compared to only 10% of the subjects in the IG, decreasing their risk of falls following the intervention.

The mean relative FAC gain was 30% higher in HG compared to IG, and the mean relative TT gain was 15% higher in HG compared to IG, suggesting a reduced risk for fall. Moreover, in the HG, 75% of the subjects increased their FAC level ≥4 after the treatment versus 55% of subjects in IG. The 0.5 to 1.5-point increase in the FAC observed in this study is in good agreement with previous studies reporting FAC increase following RAGT with Lokomat [13,16]. Our rehabilitation center considers a meaningful change in function to correspond with approximately 1 point in the FAC but it is difficult to determine with certainty if this difference could be considered as clinically meaningful since no previous studies reported values for MDC or MCID for FAC. Conesa et al. [68] consider a 2-point change in FAC as a clinically meaningful change after RAGT. Here, 40% of HG subjects had almost a 2-point increase in FAC compared to only 15% of IG subjects.

Considering the self-chosen gait velocity of the subjects, a significant increase of approximately 0.05 m s^-1^ in both groups was observed using the 10MWT results. Although small, it might be considered as a clinically-meaningful improvement [69]. This incremental improvement in speed is consistent with previous studies of the effect of 12 to 24 hours of RAGT on post stroke patients [11,13,16].

Task-specific training, like RAGT, has been recommended from a perspective of recovery of neuroplasticity [70]. Besides the fact that the subacute stroke phase recovery varies widely among individuals [71] and that the term ‘recovery’ is confusing because it is used to describe both the amelioration of neural deficits and functional improvements [72]. One major weakness of RAGT studies is the absence of consensus regarding the cut-off duration value between subacute and chronic post-stroke phases, generally between 3 and 12 months (3 months: [8,53]; 6 months: [11,15,16,73]; or 12 months: [12,74], which makes it difficult to interpret the clinical outcomes between studies. In this study, we used the cut-off value between sub-acute and chronic stoke of 3 months post stroke, that was proposed by Mehrholz et al. [8]. In the methodology of the Cochrane review on the same topic, including 23 trials and 999 participants, and showing that RAGT combined with physiotherapy may improve recovery of independent walking in people in the first 3 months after stroke but not in people after 3 months.

The participants included in this study were in subacute and chronic phases and ambulatory (FAC ≥ 1), needing the assistance of one external person to support body weight or assist balance/ coordination during walking on level surface. Another specificity of our sample was that only severe ADL-dependent subjects were included, with a low BI score (≤ 60). This inclusion criterion was motivated by the assumption that subacute stroke patients with greater motor impairments are expected to be the ideal candidates for effective RAGT [22]. In our study, we included a small number of subacute subjects (n = 9) compared to the chronic ones (n = 31) and significant functional improvements were although observed. Another assumption is that patients with no significant walking deficits, namely with FAC scores ≥ 3 may not benefit from walking in a robotic device [22]. In this study, we included subjects with various walking independence levels, including FAC scores ≥ 3, that benefits from the intervention. These differences may be explained by the severely dependent status of the chronic stroke subjects included in our study, even if some of these had no significant walking deficits estimated with the FAC.

In the endurance testing using the 6MWT, a substantial change ranged between 47 and 49 m and a small change ranges between 19 and 22 m [72]. In this study, the distance covered during the 6MWT was significantly increased in approximately 30 m in IG and 19 m in HG. Although significant intra-group improvements were observed (Table 2), our results indicate only a minor clinical change in the 6MWT following the intervention. Our results are again in good agreement with those of Hidler et al. [16] who reported a distance increase of approximately 27 m and those reported by others were slightly lower, ranging between 11 and 16 m [11,15].

Although a reduction in the median TUG timing was seen in the HG, a statistically significant improvement in TUG was observed in the IG alone, with a reduction of approximately 9 s following RAGT (Table 2). Flansbjer et al. [75] reported a standard error of measurement (SEM) value of 1.14 s, i.e. a MDC of 3.16 s and therefore this relatively important functional improvement should be considered as a clinically meaningful difference. Our findings show that 70% of IG and 55% of HG subjects had gains greater than 3.16 s, even if median gain difference was not significant in HG after RAGT.

Lokomat training parameters were optimized for each participant according to a sequence of variable progression described in the Materials and methods section. Among the training variables, specific attention was paid to the guidance force provided to assist motion of the limbs and to keep it at its minimal level. We believe that this strategy is crucial for the success of the treatment since a low level of guidance force during RAGT optimizes the involvement of the sensorimotor cortex and enables motor learning [76]. Cho et al. [77] identified chronic ambulatory-dependent patient subgroups that increased their BBS scores after RAGT: a group with a median guidance force <45% and a group with a median BWS <21%. At the end of our intervention, the median guidance force was 32.5% in HG and 30.0% in IG and median BWS was 20.0% in HG and 22.5% in IG (Fig 3), indicating that our groups were suitable to observe balance improvements.

In interpreting our findings, several limitations of our study must be considered. The first limitation is that the results are obtained from a retrospective analysis so we had no control over the data collection process. This affected our chosen matching strategy between the groups. Ideally, we would have chosen the powerful predictor of outcome for stroke, the National Institutes of Health Stroke Scale (NIHSS), with a score evaluated within 24 hours of symptom onset [78]. The NIHSS score provides a quantitative measure of stroke-related neurologic deficit. Unfortunately, the NIHSS score was not available to us. Conversely, like Kelly et al. [30], we believe that recovery after stroke described in terms of functional outcome may be more relevant to the independence of the patient than measures of neurologic deficit. Additionally, we matched our groups from the total score of the BI and not using the more specific matching utilizing sub-scores of the BI [79]. The second limitation is our small sample size in each group and that our intervention was only tested in a single clinical environment. Thus, our findings are not necessarily generalizable to a broader population. In a multicenter study design, we would have had the possibility to increase our sample and to generalize our results to a larger scale. The third limitation is that IG was mainly composed of stroke subjects with large-artery atherosclerosis and is therefore not representative of the entire ischemic population. A further study, including more subjects with small-artery occlusion, cardioembolism, and the other sub-groups of the TOAST classification [49] is necessary. The fourth limitation of this study is that anxiety was not considered, although. Bragoni et al. [80] showed that psychologic features might affect the rehabilitative outcomes of RAGT. The last limitation is the absence of a scale that belongs to a participation category domain of the International Classification of Functioning, Disability and Health (ICF) [81], e.g. the Frenchay Activities Index [82]. Most RAGT studies, however, mainly focus on improvements in the activity levels [83]. Future RAGT trials with stroke patients should assess participation category domain of the ICF to provide a more complete picture of the rehabilitation outcomes.

In conclusion, contrary to common belief of clinicians that hemorrhagic stroke survivors have better functional prognoses compared with ischemic patients, our results show that ischemic survivors could experience quasi similar improvements after an intensive treatment plan blending top-down and bottom-up approaches (top-down-up), when main possible confounders are considered. We expect that these results will: (1) serve as basic data for discussion about innovative, intensive rehabilitation protocols blending conventional physiotherapy and RAGT; (2) improve the dispersion of RAGT within rehabilitation centers; (3) promote the conduct of randomized clinical trials allowing to understand the characteristics or subgroups of stroke patients that will be the more suitable for RAGT.
